# Rodent models of post-traumatic stress disorder: behavioral assessment

**DOI:** 10.1038/s41398-020-0806-x

**Published:** 2020-05-06

**Authors:** Alexander Verbitsky, David Dopfel, Nanyin Zhang

**Affiliations:** 1 Department of Engineering Science and Mechanics, The Pennsylvania State University, University Park, PA 16802 USA; 2 Department of Biomedical Engineering, The Pennsylvania State University, University Park, PA 16802 USA; 3 The Huck Institutes of Life Sciences, The Pennsylvania State University, University Park, PA 16802 USA

**Keywords:** Depression, Physiology

## Abstract

Although the etiology and expression of psychiatric disorders are complex, mammals show biologically preserved behavioral and neurobiological responses to valent stimuli which underlie the use of rodent models of post-traumatic stress disorder (PTSD). PTSD is a complex phenotype that is difficult to model in rodents because it is diagnosed by patient interview and influenced by both environmental and genetic factors. However, given that PTSD results from traumatic experiences, rodent models can simulate stress induction and disorder development. By manipulating stress type, intensity, duration, and frequency, preclinical models reflect core PTSD phenotypes, measured through various behavioral assays. Paradigms precipitate the disorder by applying physical, social, and psychological stressors individually or in combination. This review discusses the methods used to trigger and evaluate PTSD-like phenotypes. It highlights studies employing each stress model and evaluates their translational efficacies against DSM-5, validity criteria, and criteria proposed by Yehuda and Antelman’s commentary in 1993. This is intended to aid in paradigm selection by informing readers about rodent models, their benefits to the clinical community, challenges associated with the translational models, and opportunities for future work. To inform PTSD model validity and relevance to human psychopathology, we propose that models incorporate behavioral test batteries, individual differences, sex differences, strain and stock differences, early life stress effects, biomarkers, stringent success criteria for drug development, Research Domain Criteria, technological advances, and cross-species comparisons. We conclude that, despite the challenges, animal studies will be pivotal to advances in understanding PTSD and the neurobiology of stress.

## Introduction

Post-traumatic stress disorder (PTSD) is an incapacitating chronic disorder. With a 3.9% lifetime prevalence rate worldwide and a 6.4–7.8% rate in the USA, PTSD’s health burden is substantial^1–5^. Based on the World Mental Health Surveys, 69.7% worldwide (82.7% in the USA) reported exposure to a traumatic experience. While trauma exposure is a required criterion for PTSD diagnosis, only 5.6% worldwide (8.3% in the USA) of those who experienced trauma developed the disorder^1^. This is due to numerous factors, including trauma type, variation in trauma response, social support, and endogenous factors of individuals. For adults, adolescents, and children older than six years, eight diagnostic criteria, defined in the 5th edition of the Diagnostic and Statistical Manual of Mental Disorders (DSM-5), specify measures concerning the victim’s perception of trauma and symptoms. For children six years and younger, Criteria C and D (described below) are combined, making for seven diagnostic criteria^6^.

**Criterion A:** Exposure to actual or threatened death, serious injury, or sexual violence; one or more ways (e.g., direct experience, witnessing others, learning of close family member’s or friend’s trauma, and repeated or extreme exposure to aversive details).

**Criterion B:** Intrusion; one or more symptoms (e.g., nightmares, flashbacks, intrusive thoughts, and physiological reactions to trauma reminders).

**Criterion C:** Avoidance of trauma related stimuli; one or more symptoms (e.g., avoiding thoughts, people, places, conversations, activities, objects, or situations that arouse distressing memories).

**Criterion D:** Negative alterations in cognition and mood; two or more symptoms (e.g., dissociative amnesia, emotional blunting, cognitive distortion, social withdrawal, and anhedonia).

**Criterion E:** Alterations in arousal and reactivity; two or more symptoms (e.g., irritable and aggressive behavior, reckless or self-destructive behavior, hypervigilance, exaggerated startle response, concentration problems, and sleep disturbance).

**Criterion F:** Symptom duration for over one month (Criteria B, C, D, and E).

**Criterion G:** Functional impairment (e.g., social or occupational).

**Criterion H:** Disturbance not attributable to substance effects or medical conditions.

***Specify*** **whether/if:** With dissociative symptoms (depersonalization, derealization) or with delayed expression (full diagnostic criteria not met for at least six months).

Pretraumatic, peritraumatic, and posttraumatic risk and prognostic factors affect the prevalence of the above symptoms.

**Pretraumatic factors**.

*Temperamental:* Childhood emotional problems by age 6 and prior mental disorders.

*Environmental:* Lower socioeconomic status, lower education, prior trauma, childhood adversity, cultural characteristics, lower intelligence, minority racial/ethnic status, family psychiatric history, and social support (protective).

*Genetic and physiological:* Female, younger age at time of trauma, and certain genotypes.

**Peritraumatic factors**.

*Environmental:* Trauma severity, perceived life threat, personal injury, interpersonal violence, dissociation, and being a perpetrator, witnessing atrocities, or killing the enemy (for military personnel).

**Posttraumatic factors**

*Temperamental:* Negative appraisals, inappropriate coping strategies, and development of acute stress disorder.

*Environmental:* Subsequent exposure to repeated upsetting reminders, subsequent adverse life events, financial or trauma-related losses, and social support (protective).

Like for many mental disorders, animal models play a key role in deciphering the neuropathophysiology of PTSD. Although observing humans to learn about mental disorders is effective, the major obstacles to human investigations are that PTSD is variable and seldom studied throughout disorder development. Therefore, human research focuses on populations already exposed to different uncontrolled traumatic events. Animal models overcome these barriers through the ability to longitudinally monitor PTSD development pre-trauma through post-trauma with controlled stressors. In particular, rodent models are critical to understanding PTSD induction, facilitating target identification for therapies, and testing drugs for human treatment. However, these models are simplified representations of a complex condition. PTSD is challenging to model in rodents because susceptibility, an individual’s likelihood to develop long term symptomology, is influenced by the above factors. While there is no gold standard paradigm, animal models are expected to capture PTSD symptomatology (face validity), etiology (construct validity), and treatment response (predictive validity)^7^. As such, Yehuda and Antelman defined five criteria for the face validity of translational models: (1) the stressor induces PTSD biological and behavioral responses, (2) responses are intensity-dependent, (3) biological alterations persist or progress over time, (4) biobehavioral alterations are bidirectional, and (5) responses have inter-individual variability caused from experience, genetics, or both^8^. PTSD rodent models have been previously reviewed^9–15^. This review offers a comprehensive assessment of stress model variants against multiple criteria with a focus on behavioral assays used for model validation. Although a comprehensive rodent PTSD model is challenging to develop, the demand for more effective PTSD treatment and prevention strategies drives further research. Therefore, various stress paradigms that replicate specific disorder aspects are utilized to understand the pathophysiology of PTSD. Through a survey of recent literature, this review discusses rodent PTSD models that utilize acute stress, evaluating their translational efficacies, advantages, and challenges. The review concludes by highlighting opportunities for future work in studying the neurobiology of PTSD.

## Rodent models of PTSD

Although the etiology and manifestation of psychiatric disorders are complex, mammals show biologically preserved behavioral and neurobiological responses to valent stimuli which underlie the use of rodent models of PTSD. The translational benefit of designing stress studies in rodents is supported by extensive comparative neuroanatomical studies^16–18^. Accordingly, the rodent models discussed reflect core PTSD phenotypes and vary by differences in stress type, intensity, duration, and frequency. Paradigms model the disorder by applying physical, social, and psychological stressors individually or in combination (Table 1). This review focuses on acute stressors and does not extensively address sub-chronic and chronic models because acute stressors are less likely to drive the symptom co-morbidity seen in other models. Behavioral tests mentioned in this survey are discussed further in Table 2. In literature, behavioral tests are frequently referred to as models. This can be misleading, and scientists should recognize their distinction, because tests elicit acute responses for measurement while models elicit pathology^19^.

**Table 1:** Protocol and advantages/disadvantages summary of the reviewed stress paradigms.

Animal model	Stress exposure	Control exposure	Advantages	Disadvantages	References
*Physical stress*					
Electric shock	1 day: −Inescapable 2–20 s, 1.0–3.0 mA foot/tail shocks delivered through a steel grid floor (shock duration and current depend on frequency)	1 day: −Placed in shock chamber without shock; removed from vivarium; brief handling; or undisturbed	Controllable delivery and shock parameters: current intensity, duration, number, and interstimulus interval −Reproducible context and cues −Adjustable environmental cues −Reducible stress habituation	May result in physical injuries −Not ethologically relevant −No clear protocol distinction between fear conditioning and PTSD models	^25,28^
Restraint stress	1 day: −1–2 h in a Plexiglas or wire mesh tube, restricting some locomotion	1 day: −Removed from vivarium; brief handling; or undisturbed	−Inexpensive	−May result in physical injuries −Not ethologically relevant −Uncontrollable intensity	^30^
Immobilization stress	1 day: −1–2 h in an immobilization bag (often a Decapicone) or attached to a wooden board with limbs and head in a prone position, preventing locomotion	1 day: −Removed from vivarium; brief handling; or undisturbed	−Inexpensive −More intense than restraint stress	−May result in physical injuries −Not ethologically relevant −Uncontrollable intensity	^31,35^
Underwater trauma	1 day: −1-min forced swim and 20–45 s of forced submersion in a water tank	1 day: −1-min forced swim; removed from vivarium; brief handling; or undisturbed	−Reproducible context −Ethologically relevant	−May result in physical injuries −Uncontrollable intensity	^44^
Single prolonged stress	1 day: −2-h restraint, 20-min forced swim, followed by diethyl ether anesthesia until loss of consciousness	1 day: −20-min forced swim; removed from vivarium; brief handling; or undisturbed	−Combines effects of three stressors	−May result in physical injuries −Not ethologically relevant −Uncontrollable intensity	^50^
*Social defeat stress*					
Resident-intruder social defeat	5–10 days: −Daily inescapable 5–10-min contact with a novel aggressive resident −24-h housing with a resident, separated by perforated screen (only sensory contact)	5–10 days: −24-h sensory contact with novel control; 24-h housing with same control, daily 5–10-min separation by perforated screen; 30 s in a novel defeat cage without resident; 7 min in a novel clean cage; or brief handling	−Ethologically relevant −Most intense social defeat stress	−May result in physical injuries −Predictable stress −Uncontrollable intensity: variable resident aggression −Challenging to model female and adolescent aggression −Sub-chronic stress	^65,66^
Witnessed social defeat	5–10 days: −Daily inescapable 5–15-min sensory contact during the physical social defeat of a novel intruder by a novel resident −24-h housing with a resident, separated by perforated screen (only sensory contact)	5–10 days: −24-h sensory contact with novel control; daily 30-min sensory contact in a novel cage with novel control; or brief handling	−Ethologically relevant −Does not result in physical injuries (psychological)	−Predictable stress −Uncontrollable intensity: variable resident aggression −Sub-chronic stress	^67^
Cage-within-cage resident-intruder social defeat	5–10 days: −Daily inescapable 6-h housing in a wire mesh cage inside a novel resident’s home cage (only sensory contact), without food/water −1–3 unpredictable 1-min physical contacts with a resident within the 6-h session	5–10 days: −Daily inescapable 6-h housing in a wire mesh cage inside a clean larger cage without resident	−Ethologically relevant	−May result in physical injuries −Predictable stress −Uncontrollable intensity: variable resident aggression −Challenging to model female and adolescent aggression −Sub-chronic stress	^68^
*Predator stress*					
Predator exposure stress	1 day: −5–60 min unprotected/protected inescapable exposure to a cat or ferret	1 day: −5–60 min unprotected/protected inescapable exposure to context with/without toy cat; brief handling; cage transport; or undisturbed	−Ethologically relevant	−May result in physical injuries −Uncontrollable intensity: variable predator-rodent interaction −Expensive, may require additional facilities	^92,94^
Predator-based psychosocial stress	31 days: −Days 1 (light cycle) and 11 (dark cycle): 1-h inescapable immobilization during exposure to a novel cat −Daily unstable housing conditions	31 days: −Days 1 (light cycle) and 11 (dark cycle): 1-h inescapable exposure to context without cat; brief handling; or undisturbed	−Combines effects of three stressors −Most intense predator stress	−May result in physical injuries −Not ethologically relevant −Uncontrollable intensity −Chronic stress	^101^
Predator scent stress	1 day: −5–10-min inescapable exposure to fox/bobcat urine or trimethylthiazoline on filter paper/cotton pad, cat-worn collar/cloth, ferret cloth, soiled cat litter, or rat scents/calls	1 day: −5–10-min inescapable exposure to context with a neutral odor filter paper/cotton pad/ collar/cloth, unused cat litter; or undisturbed	−Does not result in physical injuries (psychological) −Ethologically relevant: rodents use the olfactory sensory system for survival-related behaviors	−Challenging to control olfactory cues (e.g., dosage of scent) −Olfaction is variable because it is driven by perception	^112,113^

**Table 2:** Rodent behavioral tests outlined by DSM-5 criteria for PTSD.

Behavioral test	Description	Measures	References
*Criterion B*	*Intrusion*		
Contextual and cued trauma reminders	Physiological and behavioral reactions to exposure arena	Heart rate, body temperature, systolic blood pressure, diastolic blood pressure, plasma corticosterone, time freezing, total locomotor activity	^25,224^
*Criterion C*	*Avoidance*		
Elevated plus-maze	Elevated platform arranged in a + with four perpendicular opposite arms: two open, two closed	% time spent in open arms, % entries made into open arms	^225^
Elevated T-maze	Elevated platform arranged in a T with three perpendicular opposite arms: one open, two closed	Latency to leave enclosed arm, latency to enter enclosed arm	^226^
Elevated zero-maze	Elevated circular platform with two opposite open and closed quadrants	% time spent in open arms, frequency of head dips over edge of platform, frequency of stretched-attend postures	^227^
Light-dark box	Two-compartment box: one white and illuminated, the other black and dark. A small opening connects the compartments	Time spent in light, number of light entries, % distance in light, number of rears, latency to enter light, number of transitions	^228,229^
Open field	Square, rectangular, or circular enclosure that is bare or covered with a thin layer of bedding	Time spent in central squares, latency to enter central squares	^230^
Novelty suppressed feeding	Open field with novel food (e.g., sugar puffs, fruit loops) in an illuminated central area	Latency to eat	^231^
Hole board	Elevated and open-topped square box with evenly spaced holes in the floor	Latency to head dip, number of head dips, number of rears, time active, time spent in center	^92^
Modified hole board	Open field with a hole board in the middle. The hole board consists of staggered holes covered by removable lids	Latency to board entry, number of board entries, % time spent on board	^232^
Conditioned taste aversion	Conditioned stimulus (e.g., novel taste of saccharin) is paired with an unconditioned stimulus (e.g., lithium chloride injection that results in nausea, stress and re-stress). Recipients avoid the new taste when it is subsequently presented	Saccharin/cyclamate intake	^233^
Conditioned object avoidance	Conditioned stimulus (e.g., plastic prism) is paired with an unconditioned stimulus (e.g., electric footshock). In separate tests, a plastic prism or cube (novel object) is placed into the home cage	Time spent with familiar/novel object, time spent burying familiar/novel object, total locomotor activity and/or sniffing floor/walls	^234^
Conditioned odor avoidance	Conditioned stimulus (e.g., odor of ethanol) is paired with an unconditioned stimulus (e.g., electric footshock). Three-compartment box interconnected by guillotine doors. Center (nest) compartment: filter paper-lined Petri dish with home-cage bedding, cleaned with soapy water. Left/right compartments, counterbalanced: Petri dish with ethanol or acetate (novel neutral odor) solutions, cleaned with respective solutions. Habituation phase: rodent in nest compartment. Test phase: free exploration	Latency to first exit from nest compartment, time spent in nest/ethanol/acetate compartment	^235^
Conditioned odor active avoidance	Conditioned stimulus (e.g., odor of acetic acid) is paired with an unconditioned stimulus (e.g., electric footshock). Two-compartment box: start compartment cleaned with acetic acid solution, the other with ethanol solution (novel neutral odor)	% time spent in acetic acid compartment, number of rears toward the acetic acid compartment	^236^
Territory discrimination	Two boxes linked to a starting box with litter covering the floor. The tested animal and a novel conspecific stay in separate compartments (personal and unknown) for 24 h before testing	Time spent in personal/unknown compartment, number of entries to personal/unknown compartment	^237^
*Criterion D*	*Negative alterations in cognition and mood*		
Morris water maze	Black circular tank filled with water in a room with visual cues on walls. The tank is conceptually divided into four quadrants and four start locations (N, S, E, W). Training sessions: animals locate or are guided to a small submerged platform (target quadrant). Probe trial: platform is removed to assess memory of its location	Time spent in target quadrant, time spent in opposite quadrant	^238^
Radial arm water maze	Black circular tank filled with water. The tank contains 4–12 V-shaped inserts that produce swim arms radiating from an open central area. Training trials: animals locate or are guided to a small submerged platform at the end of one arm (goal arm). Test trial: platform is removed to assess memory of its location	Number of arm entry errors	^239^
T-maze continuous alteration task	Elevated or enclosed platform arranged in a T with three perpendicular opposite arms: two identical goal arms and one longer start arm. Arms are separated by guillotine doors and a central partition extends into the start arm. Testing consists of one forced trial and several free-choice trials	% alternation rate	^240^
Novel object recognition	Open field with two objects at opposite and symmetrical corners. Habituation phase: no objects. Familiarization phase: two identical objects. Test phase: one familiar, one novel object	Time spent with familiar/novel object, number of familiar/novel object entries, discrimination index, index of global habituation, recognition index, preference index	^241^
Y-maze spontaneous alternation test	Platform arranged in a Y with three identical arms at 120° from each other	% alternation rate	^242^
Y-maze recognition memory test	Platform arranged in a Y with three identical arms at 120° from each other. Acquisition phase: access to two arms, one arm closed (novel arm). Retrieval phase: access to all arms	Time spent in familiar/novel arms, discrimination index	^243^
Barnes maze	Circular table with equally spaced holes around its circumference. The table’s surface is illuminated and a box is under the target hole. Acquisition trials: animals locate or are guided into the target hole. Reversal phase: target hole is moved 180° across the maze. Probe trial: target cage is removed to assess memory of its location	Latency to enter the target hole, distance traveled to the target hole, number of errors, number of entries into the former target hole during reversal/probe	^244,245^
Cued and contextual fear conditioning / Fear extinction	Operant chamber in a ventilated, sound-attenuated cubicle with a shock grid floor, speaker, and video camera. An unconditioned stimulus footshock is paired with a conditioned stimulus tone or context. Fear conditioning involves longer intertrial intervals and fewer stimuli than fear extinction training/testing	Time freezing	^246,247^
Differential contextual odor conditioning	Cue (cinnamon odor) signals reward or punishment depending on the context. Appetitive conditioning (arena 1): cue + reward (sweetened water). Aversive conditioning (arena 2): cue + conditioned cue (tone) + aversive unconditioned stimulus (electric shock). Testing (arena 3): cue	% time freezing	^248^
Step-through inhibitory (passive) avoidance	Two-compartment box: one white and illuminated (safe), the other black and dark (unsafe). A sliding door connects the compartments. Training phase: door closes upon entrance to the dark compartment and an unconditioned stimulus footshock is delivered. Testing phase: no shock	Latency to enter the dark compartment, time spent in dark compartment	^249^
Response bias probabilistic reward task	Tone discrimination training: operant testing chamber with two levers, a food receptacle, and a speaker. Rodents discriminate between two tone stimuli by pressing an associated lever. Testing: ambiguous tone durations. Correct identification of one tone (rich stimulus) is reinforced with a food pellet three times more frequently than the other tone (lean stimulus)	Response bias, discriminability, accuracy (% correct), reaction time	^250^
Forced swim test (Porsolt forced swim test or behavioral despair test)	Rodents are placed inside a cylindrical water tank	Time immobile	^251,252^
Tail suspension	Rodents are hung by their tails	Time immobile	^253^
Open field	Square, rectangular, or circular enclosure that is bare or covered with a thin layer of bedding	Total locomotor activity, ambulatory locomotor activity, distance traveled, frequency of rearing, frequency of sniffing	^254^
Flinch-jump test	Operant chamber in a ventilated, sound-attenuated cubicle with a shock grid floor, speaker, and video camera. Shock titrations are delivered in a series of ascending and descending intensities based on the rodent’s response	Flinch threshold, vocalization threshold, jump threshold	^53^
Hot-plate test	Rodents are placed in a glass beaker on a plate heated to a constant temperature	Latency to flinch or raise hind paws	^255,256^
Tail flick test	The distal third of a rodent’s tail is thermally stimulated with radiant heat (e.g., focused light from a light bulb), immersion in hot water, or direct contact with a heated surface	Latency to withdraw tail	^257^
von Frey test	von Frey filaments are applied to the plantar region of a rodent’s paw through a wire mesh floor. Stimulation continues in a series of ascending and descending filament forces based on the rodent’s response	Paw withdrawal threshold	^258^
Sucrose preference	After habituation to two drinking bottles, one water bottle is replaced with a 1–2% sucrose solution. Bottle positions (left vs. right) are alternated each day to control for side-preference bias	% sucrose preference, % sucrose intake, % water intake	^259^
Intracranial self-stimulation	Operant chamber in a ventilated, light-attenuated and sound-attenuated cubicle with a wheel or lever manipulandum on the wall. Bipolar electrodes are implanted into a brain region that is part of the reward system. Training: manipulandum response following noncontingent stimulation prompts an identical contingent stimulation. Testing: stimulations are delivered in a series of ascending and descending intensities based on the rodent’s response	% baseline current-intensity threshold, response latency, number of extra responses, number of time-out responses	^260,261^
Social interaction test	Two novel conspecific rodents (same sex, size, and age) interact freely in an open field	Time spent in active social interaction (sniffing, licking, close following, allogrooming, crawling over the partner), number of social interactions, time spent in social avoidance (escaping, keeping the partner at distance with upright forepaws)	^262^
Social preference/avoidance test	Open field with a wire cage centered against a wall of the arena. One trial without a rodent in the cage (no target) and one trial with a rodent in the cage (target)	Time spent in the interaction zone with the target absent/present, time spent in the two corner zones opposite the wire cage, social avoidance ratio	^263,264^
Partner preference test	Two boxes linked to a starting box. One female and male rodent is tethered to the rear of a goal box	Time spent with male/female, time spent in active social interaction with male/female, number of visits to male/female	^265^
Social approach/avoidance test	Two chambers of different widths connected by a sliding door. A large conspecific rodent (stimulus) is enclosed in a compartment of the large chamber, separated by a transparent perforated wall. The sliding door is removed after a habituation phase in the smaller chamber	% time spent in the large chamber compartment, number of entries to the large chamber	^266^
Three-chamber sociability and social novelty test (Crawley test)	Three-chambered box connected by sliding doors and with one wire cage in each side chamber. Habituation trial: rodent is placed in the center chamber with obstructed access to side chambers. Sociability test: novel conspecific rodent (same sex, size, and age) is placed in one of the wire cages. Social novelty test: another novel conspecific rodent is placed in the opposite cage	Time spent in empty chamber, time spent in social chamber 1, time spent in social chamber 2, social interaction ratio, social novelty preference index	^267^
Olfactory habituation and dishabituation test	Glass slides with drops of water (nonsocial odor) and two social odors of diluted urine from different same-sex rodents are sequentially presented into a cage. Three consecutive trials of each odor are conducted	Time spent in direct olfactory investigation (sniffing)	^268^
*Criterion E*	*Alterations in arousal and reactivity*		
Object burying	An unfamiliar object is placed on the surface of bedding in a cage	% time spent manipulating object, % time spent burying object	^224^
Marble burying	10–20 glass marbles are spaced evenly on the surface of bedding in a cage	Number of marbles buried (to 2/4 their depth), latency to dig, time spent digging, rearing count	^32,269^
Acoustic startle response	Cylindrical animal enclosure on a platform inside a ventilated, sound-attenuated cabinet. Speakers produce a continuous background noise and the acoustic stimuli. An acclimation period is followed by startle noise trials	Startle amplitude, average startle response	^97,270^
Prepulse inhibition	Cylindrical animal enclosure on a platform inside a ventilated, sound-attenuated cabinet. Speakers produce a continuous background noise and the acoustic stimuli. An acclimation period is followed by pulse-alone trials, prepulse + pulse trials, and no stimulus trials in pseudorandom order	Startle amplitude, % prepulse inhibition	^271^
Operant attentional set-shifting tasks	Operant chamber in a ventilated, sound-attenuated cubicle with two operandi (e.g., nose-poke holes or levers) on each side of a food dispenser and yellow lights. Correct responses are reinforced with palatable food (e.g., sucrose solution or sucrose pellets). Pretraining: rodents associate stimuli with reward. Response discrimination reversal-Response discrimination training: rodents respond on the operandum opposite to their side bias. Response discrimination reversal: rodents respond on the opposite operandum. Visual-cue-to-place set-shifting-Visual-cue discrimination training: rodents respond on the illuminated operandum. Shift to response discrimination: rodents respond on the operandum opposite of their side bias, regardless of the light’s position	Number of errors to criterion, number of errors over 20 trials, total number of trials to criterion, number of error trials, number of errors by type (pervasive, regressive, never-reinforced)	^272,273^

### Physical stressors

Physical stressors used to develop PTSD models include electric shock, underwater trauma, restraint/immobilization stress, and single prolonged stress. These stressors are advantageous for their procedural simplicity, clear symptom impact, and ease of scaling. Although widely utilized, physical stressors have not reproducibly differentiated individual variability or sex differences because most subjects display behavioral consequences. Physical stressors can also elicit injuries, pain, and/or inflammatory responses that can confound behavioral test results, including measures of motivation and movement^20^.

#### Electric shock

Electric shock is employed in animal models of anxiety, depression^21^, and PTSD. Inescapable and unpredictable electric shock can be administered through the animal’s tail or foot via a steel grid floor in a shock chamber. These studies combined high-intensity currents (1.0–3.0 mA) with long durations (2–20 s) to induce lasting symptoms. Currents and durations were typically larger than those applied in fear conditioning (0.5–1.5 mA, 0.5–2 s) aiming to investigate short-term fear learning. Currently, learning and trauma protocols are not clearly differentiated as there is no clear evidence for separating “nontraumatic” shocks that are within a rodent’s coping capacity from “traumatic” shocks that are beyond its coping capacity^22^. However, this stressor is more frequently used to study learning and memory, its initial application, rather than to model PTSD. Electric shock may be combined with contextual and/or cued trauma reminders (associative fear) through re-exposure to shock chambers and conditioned stimuli, and with neutral stimuli in a novel environment (non-associative fear). In stress-enhanced fear learning, pre-exposure to repeated intense footshocks in context A increased rodent fear response in context B when only a single shock, a less intense stressor, was administered^23^. This non-associative sensitization effect elevated freezing in context B for three months, even after context A extinction training, reflecting resistance to exposure therapy (extinction)^24^. Upon similar situational reminders, rodents exhibited behavioral responses comparable to human PTSD intrusion symptoms, such as crouching against the chamber wall, as well as increased freezing, respiratory rate, and fecal boli^25–27^. Since re-exposure to stressor-related environments/cues is the clinical analog of exposure therapies, the electric shock model can be studied to advance cue-based out-of-context (reminders in office) and in-context (virtual reality) therapies^14^. Advantages of electric shock as a PTSD model are its controllable delivery and shock parameters (current intensity, duration, number, and interstimulus interval), reproducible context and cues, adjustable environmental cues, and reducible stress habituation^28^. For review of electric shock, see Aliczki and Bali & Jaggi^22,29^.

#### Restraint/immobilization stress

Restraint and immobilization stresses include confining rodents in enclosed chambers to limit movement for an extended period of time. Restraint stress is generally conducted by placing animals in Plexiglas or wire mesh tubes^30^. Immobilization stress is achieved by either placing animals into rodent immobilization bags (often Decapicones)^31–33^ or attaching the animal’s limbs and head in a prone position to wooden boards^34,35^. Currently, restraint and immobilization protocols are not clearly differentiated and the two terms are often used interchangeably^36^. While restraint and immobilization are similar in that they are stressors driven by the limitation of motion, it is important to make the distinction between the two as restraint does not prevent, but only restricts, movement of the rodent’s limbs, body, and head. Accordingly, immobilization elicits stronger responses than restraint^37^. There are no comparative studies favoring any one acute stress protocol. Furthermore, only two studies comparing the sex-dependent effects of immobilization stress have been reported. One study found that the attribution of incentive salience toward food reward location (goal-tracking) was increased in Long-Evans males exposed to acute immobilization stress, whereas a bias toward food reward-associated cues (sign-tracking) was increased in females. This suggests sex-differences in the failure to appropriately assign motivational value as related to amotivation/anergia and anhedonia^38^. Another study, in agreement with one subgroup of human endocrine responses^39,40^, reported that immobilization stress evokes long-term hypothalamic-pituitary-adrenal (HPA) axis desensitization following re-exposure to the same (homotypic) stressor and sensitization to novel (heterotypic) stressors^35^. This effect was greater in female Sprague-Dawley rats^41^. Studies also noted delayed expression (10 days post-stress) of avoidance behavior and changes in dendritic spine density in the basolateral amygdala, a stress responsive brain region^31^. For review of restraint and immobilization stress, see Buynitsky^42^.

#### Underwater trauma

Underwater trauma, or submersion stress, involves 1 min of forced swim followed by 30 s of forced submersion in a water tank. Exposed rats demonstrated immediate and persistent (7–30 days post-stress) increased arousal in acoustic startle response (ASR) and anxiety-like behavior in the elevated-plus maze (EPM) tests compared to control rats that swam without submersion^43–45^. Learning deficits in Morris water maze (MWM) spatial memory tasks, observed three weeks after trauma exposure, demonstrated the stressor’s lasting negative effects on cognition^44^. However, interpretation of MWM results can be confounded by the re-exposure to water, a reminder of underwater trauma, that may influence task performance. Decreased plasma basal corticosterone (CORT) levels were found seven days after stress, indicating a lasting depression of HPA axis signaling following the trauma^43^. Based on successive performance in EPM and ASR, cut-off behavioral criteria demonstrated that the prevalence of maladaptive response to stress dropped from 91.6% of exposed Sprague-Dawley rats on day 1, the acute phase, to a constant rate of 41.6% by day 7 through day 30. Concomitantly, the prevalence of well-adapted rats rose from 0% to 25% over days 1–30^45^. This temporal pattern reaffirms that animals display an individual variation in response similar to humans^46^. That is, the initially large affected proportion of the exposed population decreases steadily with time as many individuals show a tendency toward symptom improvement. Some may present acute stress disorder and a minority develop PTSD. Behavioral profiling, validated by immunohistochemical assessments, uncovered three separate stress response phenotypes: an anxious, fear-based group (38%), a co-morbid, fear-anhedonic group (15%), and an exposed-unaffected group (47%). In accordance with the high anxiety trait of the posttraumatic depression model^47^, enhanced pretrauma freezing correlated with posttrauma saccharin preference among fear-anhedonic phenotype rats, predicting anhedonia one month after exposure^48^. Although its intensity cannot be easily controlled, advantages of underwater trauma as a PTSD model are its reproducible context and ethologically relevant stress.

#### Single prolonged stress

Single prolonged stress (SPS) involves sequential administration of three stressors—2-h restraint, 20-min forced swim, and diethyl ether anesthesia—with a 7-day or 14-day undisturbed sensitization before testing. According to time-dependent sensitization studies, the undisturbed incubation period is necessary for PTSD-like symptom manifestation^49^. Interestingly, studies observed most behavioral and cellular changes seven days after SPS or re-exposure, indicating time-dependent and experience-dependent sensitization^50^. Consistent with findings in PTSD patients, neuronal apoptosis and autophagy dysregulation in the hippocampus, amygdala, and prefrontal cortex appeared one day after stress, suggesting that morphological changes precede behavioral alterations^51^. Combination of the three stressors is required for the PTSD phenotype, as combinations of any two does not induce all effects observed in the SPS paradigm^52^. As in underwater trauma, interpretation of forced swim and MWM test results is confounded by the re-exposure to water. Although age effects on SPS susceptibility have not been evaluated to date, maternal separation was found to strengthen adult SPS-induced increases in anxiety and contextual fear^53^. Early life exposure to SPS caused anxiety-like and depression-like behavior at postnatal day 32 (human early-adolescence), anxiety-like behavior at postnatal day 60 (human late-adolescence), followed by depression-like behavior (stress-susceptible) or no behavioral deficits (stress-resilient) at postnatal day 90 (human adulthood), suggesting that adaptations such as behavioral and cognitive switching occur at postnatal day 60^54^. The two studies of sex differences in fear extinction retention following SPS report conflicting results, with one observing no effect^55^ and the other noting deficits^56^. The finding that female rats express fear by darting rather than freezing indicates that freezing alone may be misleading and motivates reinterpretation of female rodent fear conditioning studies^57^. SPS increased the latency of pair-housed Sprague-Dawley females to approach a novel rat in the social preference/avoidance test, implying an anxious phenotype, but decreased the latency of single housed females, implying social support seeking^58^. The dexamethasone suppression test revealed an exaggerated negative feedback control of the HPA axis in SPS-exposed Sprague-Dawley males, but not in females^59^. SPS lacks ecological validity and its stressor intensity cannot be modified. However, an advantage of SPS as a PTSD model is its combination of stressors to produce a synergistic effect. For an extensive review of SPS, see Lisieski, Souza, and Yamamoto^60–62^.

### Social and psychological stressors

Social and psychological stressors include social defeat and predator stress. Advantageous for their ecological validity and relevance to interpersonal assault traumas^63^, these stressors are widely utilized in differentiating individual variability. While predator stress can be conducted on both sexes, social defeat is challenging to model in female rodents because they tend not to defend territories with aggressive behavior. When using direct exposure to a resident rodent or predator, reproducibility of the stressor can be challenging due to variation in rodent and predator aggression. If the social and psychological nature of these stressors causes physical injuries, behavioral measures may then reflect physical rather than neurological driven effects.

#### Social defeat stress

Social defeat stress (SDS) is used in rodent models of depression^64^ and PTSD. There are three variations of SDS: resident-intruder, witnessed social defeat stress (trauma witness or vicarious social defeat), and cage-within-cage resident-intruder. While in the resident-intruder paradigm, an experimental rodent (intruder) is exposed daily (5–10 days; 5–10 min per day) to a novel dominant conspecific (resident)^65,66^, witnessed social defeat stress is induced by daily inescapable sensory contact with novel rodents undergoing physical social defeat^67^. In both procedures, subordination may then be reinforced through 24 h of sensory (visual, olfactory, and auditory) contact with the resident, separated by perforated screen. The cage-within-cage resident-intruder paradigm introduces rodents to sensory contact with novel residents for 6-h sessions that include one to three 1-min unpredictable physical contact periods^68^. Since territoriality is established in sufficient living space and enhanced in the presence of a sexual partner and sexual experience, the intruder is either introduced to an individually housed resident or replaces a cohabitating partner in the resident cage^69^. Intruders with lower body weights than residents are frequently used to guarantee intruder defeat^66^. Aggressive behaviors, different between male and female rodents, are characterized by attacking, pushing, aggressive grooming, chasing, pinning, and upright or lateral dominant postures^70^. Depression models employ chronic SDS, with stress repeated for 10 days to five weeks. It has, however, been suggested that chronic SDS may not only be a depression model, but may be more appropriately used to simulate the depression, anxiety, and social avoidance dimensions of PTSD^71^.

Male rodents exposed to resident-intruder social defeat manifest a persistent behavioral syndrome. The social preference/avoidance test categorized 50–70% of exposed C57BL/6 J mice as susceptible to stress^65,72^. Interestingly, social rank predicted this individual variability through a link between response strategy and outcome, showing that dominant mice were more susceptible than subordinate mice^73^. Preexisting individual differences in the peripheral immune system also predicted stress susceptibility^74^. Morning CORT levels showed no changes among groups at day 11, but decreased in susceptible mice and increased in resilient mice at day 39 after social defeat^72^. In another study, stress resilience was associated with the emergence of the gram-positive bacteria Bifidobacterium. This finding is relevant to clinical applications as gut microbiota dysbiosis is found in patients with PTSD^75^. Many studies have also shown that environmental enrichment promotes adaptive behavior and brain function. Enriched environmental housing before social defeat conferred stress resiliency. Further investigation, through lesion of the infralimbic cortex, illustrated that the ventromedial prefrontal cortex was involved in the acquisition of protective effects^76^. Extending this observation, optogenetic modulation of neuron projections to/from the ventromedial prefrontal cortex^77^, ventral tegmental area^78,79^, nucleus accumbens^80^, and dorsal raphe nucleus^81^, key nodes of PTSD circuitry, exerted antidepressant-like effects in susceptible mice.

Female-female agoniztic behaviors show low levels of direct attack in resident-intruder confrontations compared to inter-male aggression, limiting studies exploring the effects of social defeat in female rodents^70^. Furthermore, male behavior towards female versus male intruders is not similarly motivated and results in fewer attacks. As such, resident-intruder pairings are frequently same-sex conspecifics. The rarity of female aggressive behavior has been addressed through using lactating dams^82^, aggressive species such as California mice and Syrian hamsters^83,84^, mediobasal hypothalamic lesions^85^, as well as testosterone treatment in neonatal^86^ and ovariectomized adult female rodents^87,88^. Female CD-1 mice exposed to lactating dams displayed elevated anxiety in the EPM, with more pronounced effects two weeks, but not 2 h, after social defeat^71^. Although qualitatively different from male SDS, recent studies have induced male aggression toward females through application of male odorants on females^89^ and chemogenetic activation of the ventrolateral subdivision of the ventromedial hypothalamus in males^90^. The limited number of female rodent studies using similar species, methodologies, and behavioral tests complicates result comparison. In addition, cross-sex comparison is contingent upon proof of equivalent attack magnitude toward males and females. While care is taken to minimize severe injuries, behavioral measures may be compromised when the social nature of resident-intruder and cage-within-cage resident-intruder paradigms is mixed with the stress of physical injury. SDS is also limited by the potential for adaptation to its predictable repeated stress. Although the stressor type cannot be varied, its predictability could be reduced by exposing rodents to novel residents at different times each day for different stress durations. For review of social defeat, see Hammels^91^.

#### Predator stress

Predator stress models, prioritizing ecological validity, expose rodents to species-relevant predators or their scents. Variations of this stressor include predator exposure stress, predator-based psychosocial stress (PPS), and predator scent stress (PSS).

In the predator exposure stress model, rodents are acclimated for 5 min in an inescapable exposure environment, followed by a 5–60 min exposure to an unprotected/protected (subject is free/caged) cat^92,93^ or ferret^94^. While initial acute cat exposure studies assessed overall behavior through tests of risk assessment, anxiety, and arousal^92^, later reports also found avoidance of trauma-reminder in the open field test^95^, with spatial memory retention impairments in the MWM and radial-arm water maze^93,96^. Like SPS, exposure enhanced dexamethasone suppression of CORT in Sprague-Dawley males, but not in females^59^. In addition, acute ferret exposure in Sprague-Dawley rats produced sensorimotor gating abnormalities in prepulse inhibition^94^. Out of the exposed Sprague-Dawley rats, 25.3% developed PTSD-like behavioral and endocrine dysregulation^96^. Sex differences in vulnerability were test specific, noted in EPM, open field, and ASR measures. Sex differences were also identified in hole-board and light-dark box measures which were unaffected by exposure^95,97^. Persistent behavioral changes, some lasting at least three weeks after exposure, corresponded to changes in the amygdala, medial prefrontal cortex, and hippocampus. These structural and molecular changes are consistent with brain regions involved in PTSD^93,95,98,99^. However, given predator exposure’s variable cat-rodent interaction and the challenge of controlling cat aggression, both cat and rodent behavior must be assessed. For review of predator exposure, see^100^.

The PPS model, composed of acute and chronic components, combines 1 h of acute immobilization during novel cat exposure on days one (during the light cycle) and 11 (during the dark cycle), with 31 days of chronic unstable housing conditions to produce a risk factor synergistic effect. Following observations that rodents directed their postures away from cats, providing them with an element of control over confrontations, immobilization was included in this model as an analog to the sense of helplessness prominent in PTSD. Repeated cat exposure was included to (1) apply to people who develop PTSD only after multiple traumas; (2) mimic intrusive trauma reminders by forcing rodents to re-experience the original stress; (3) mimic the unpredictability of re-experiences (light or dark cycle); and (4) augment stress-induced changes. Further increasing symptom prevalence, daily randomized housing conditions were included to mimic chronic mild stress and lack of social support^101^. Three weeks after the second predator exposure, all stressed Sprague-Dawley rats exhibited PTSD-like sequelae. PPS also caused reduced growth rate, thymus weight, and basal glucocorticoid levels, as well as increased adrenal gland weight and physiological reactivity to an acute stressor^101–103^. Some effects were still observed more than four months after stress onset^104^. Neurotransmitter changes, such as greater norepinephrine and reduced serotonin levels in the hippocampus and prefrontal cortex, are in concert with human PTSD research^105–107^. This paradigm was also shown to induce sex-dependent cardiovascular alterations with notable male and ovariectomized female increases in myocardial sensitivity to ischemic injury. Stressed female rats displayed anxiety-like behavior in the EPM and open field, irrespective of estrous stage or ovariectomy condition. However, stressed female rats did not demonstrate physiological effects other than a reduced growth rate^108,109^. For review of PPS, see Zoladz^110^.

The PSS model involves a 5 to 15-min inescapable exposure to fox/bobcat urine^111^, soiled cat litter^112^, a cat-worn collar/cloth^113^, a ferret cloth^114^, or trimethylthiazoline (TMT), a synthetic compound isolated from fox feces^115^. Similar findings in successive ferret and cat cloth exposures confirmed failure of stress-habituation^114,116^. Moreover, different amounts of TMT^117^ and sizes of cloth impregnated with cat scents^118^ elicited fear-related behavior in a dose-dependent manner. After application of median split criteria, the incidence of susceptible TMT-exposed Sprague-Dawley rats ranged from 14 to 21.8%^119^. Similarly, after cut-off behavioral criteria, extreme behavioral responses (PTSD-like) to soiled cat litter were observed among 50% of Lewis, 10% of Fischer F344, and 25% of Sprague–Dawley rats^112^. Although sensitive to strain differences, cut-off behavioral criteria did not distinguish PTSD prevalence among male and female Sprague-Dawley rats^120^. In both sexes, extreme behavioral response rats displayed inhibited cardiac autonomic system habituation and recovery after exposure^121^. Early life stress increased vulnerability to cardiac autonomic dysfunction, and blunted basal CORT pulse amplitude predicted post-exposure PTSD susceptibility^122,123^. Avoidance of bobcat urine-paired context predicted post-stress thermal hyperalgesia in Wistar rats with high stress reactivity^111^. Interestingly, resting-state functional magnetic resonance imaging (rsfMRI) noninvasively detected prolonged neuroadaptation within the amygdala-medial prefrontal cortex circuit in Long-Evans rats exposed to cat collar^113^. Another study found that pre-existing functional connectivity in olfactory and stress-related neural circuits might predispose animals to differential stress responses, linked to PTSD susceptibility, during fox urine exposure. Susceptible rats exhibited less freezing, but greater avoidance of fox urine, and displayed a prolonged CORT response, as well as higher anxiety long after exposure^124^. These findings indicate the importance of analyzing behavior during exposure. A high-dose of CORT injected subcutaneously 1 h before soiled cat litter exposure reduced the prevalence of extreme behavioral responses from 50% to 8% in Lewis rats, indicating that elevated CORT levels before acute stress prevent later stress effects^112^. Analogous to clinical trial results, high-dose CORT administered 1 h after stress reduced the prevalence of PTSD-like rats, reversing extreme behavioral disruptions along with molecular and morphological measures in the hippocampal dentate gyrus. This evidence supports the use of high-dose CORT in trauma care and suggests that there is a treatment “window of opportunity” early after trauma^125^. Combined, these observations indicate the protective effects of glucocorticoids against the development of PTSD. Sleep deprivation for 6 h during the first resting phase after PSS attenuated PTSD-like behaviors in EPM, ASR, and hippocampal expression of glucocorticoid receptors, demonstrating an avenue for secondary prevention of stress-related clinical disorders^126,127^. Since predator scent can impregnate testing rooms and influence control animals, careful handling and stressing under a fume hood is advised. Olfactory cues such as dosage of a scent are challenging to control because olfaction is variable and driven by perception. An advantage of PSS as a PTSD model is its ecologically relevant stress, as rodents use the olfactory sensory system for survival-related behaviors^128^. For review of PSS, see Cohen and Staples^129,130^.

### Translational studies of PTSD

Established stress paradigms that induce PTSD-like behavioral and biological phenotypes are available (Table 1). However, since stressor severity varies, stressful experiences that are within a rodent’s coping capacity should be distinguished from traumatic experiences that are beyond its coping capacity. Rodent behavioral tests, mimicking the tests conducted in humans, are used to assess stress effects and allow researchers to make inferences about rodent psychology (Table 2). Although all stress models produce lasting general anxiety or depression effects, variety in behavior robustness makes each paradigm effective at targeting specific constructs. Behavioral tests and neurobiological changes are used to evaluate an animal model’s representation of the human disorder, satisfying DSM-5 PTSD symptom clusters (Tables 3, 4), validity criteria (Table 5), and Yehuda and Antelman’s criteria (Table 6). Unlike for the DSM-5, no criteria have been established to assess how well an animal model meets validity or Yehuda and Antelman’s criteria. Gaps in literature were addressed as no one model has been proven to satisfy all criteria.

**Table 3:** Evaluation of reviewed animal models against DSM-5 criteria for PTSD–effects in males^6^.

	Criterion B: intrusion	Criterion C: avoidance of trauma related stimuli	Criterion D: negative alterations in cognition and mood		Criterion E: alterations in arousal and reactivity			Criterion F: lasting symptoms
	Physiological reactions to trauma reminders	Increased avoidance	Cognitive alterations	Mood alterations	Increased arousal	Concentration problems	Sleep disturbance	Symptoms present
***Physical stress*** Electric shock	Contextual and cued reminders^25,224^	EPM, OF, CODA, conditioned object avoidance, NSF, modified hole board^28,234,235,274^	MWM^275^	Tail flick, SAAT, SI, FST^27,28,266^	Object burying, ASR^25,224^		EEG^234^	>Month^24,276^
Immobilization stress	Contextual reminder^34,35^	EPM, OF, hole board, mirror chamber, LDB^31,34,277–279^	FCFE, MWM, NOR, Y-maze spontaneous alteration test^34,280,281^	Incentive salience, SP, FST^38,278,282^	ASR, MB^32,283^		EEG, EMG, EOG^33^	>1 Week^31^
Underwater trauma	Contextual reminder^48^	EPM, hole board, OF^43,284,285^	DCOC, MWM^44,248^	SP^48^	ASR^45^			> 3 Weeks^44^
Single prolonged stress	Cued reminder^286^	Conditioned taste aversion, OF, EPM, elevated T-maze, LDB, cliff avoidance^53,54,287–290^	NOR, FCFE, RAWM, MWM^51,52,54,291,292^	Three-chamber sociability and social novelty test, flinch-jump, hot-plate, SP, FST, von Frey^53,287,292–294^	ASR, MB^295,296^	Set-shifting^273^	EEG, EMG^297^	>Month^54,289,293^
***Social defeat stress*** Resident-intruder social defeat	Contextual reminder^20^	EPM, LDB, EZM^72,73,298^	Response bias probabilistic reward task, MWM, Barnes maze, step-through inhibitory avoidance, FCFE, RAWM, NOR, Y-maze recognition memory test, T-maze continuous alteration task^299–306^	SPAT, three-chamber sociability test, intracranial self-stimulation, SAAT, SP, FST, OF, tail suspension, olfactory habituation-dishabituation^76,264,266,307–310^	ASR^311^		EEG, EMG^312^	>Month^72^
Witnessed social defeat	Contextual reminder^20^	EPM, LDB^67,313^	RAWM^313^	Three-chamber sociability test, OF, SPAT, SP, FST^67,313,314^				>Month^315^
Cage-within-cage resident-intruder social defeat	Contextual reminder^68,316^	Partition test^68^	Y-maze spontaneous alteration test^242^	FST, tail suspension^317^				>Month^68^
***Predator stress*** Predator exposure stress	Cued reminder^95^	EPM, hole board, LDB, elevated T-maze, OF^92,95,97,318^	MWM, RAWM^93,96^	SI^92^	ASR, PPI^94,97^			>3 Weeks^92^
Predator-based psychosocial stress	Contextual and cued reminders^102^	EPM^101^	NOR^101^		ASR^101^			> Month^104^
Predator scent stress	Contextual reminder^111,119,319^	EPM, territory discrimination, LDB, OF, hole board^112,116,129,320,321^	MWM, DCOC, NOR^115,248,322^	Partner preference, Hargreaves test, SI^111,116,129^	PPI, MB^112,124^		EEG, EMG, LFP^319^	>Month^122^

**Table 4:** Evaluation of reviewed animal models against DSM-5 criteria for PTSD—Effects in females^6^.

	Criterion B: intrusion	Criterion C: avoidance of trauma related stimuli	Criterion D: negative alterations in cognition and mood		Criterion E: alterations in arousal and reactivity			Criterion F: lasting symptoms
	Physiological reactions to trauma reminders	Increased avoidance	Cognitive alterations	Mood alterations	Increased arousal	Concentration problems	Sleep disturbance	Symptoms present
***Physical stress*** Electric shock	Contextual reminder^26,276,323^	EPM, LDB^276,323^		SI^323^				> Month^276,323^
Immobilization stress	Contextual reminder^41^			Incentive salience^38^				>1 Week^41^
Underwater trauma								
Single prolonged stress		EPM^324^	FCFE^56^	FST, SP, SPAT, von Frey^58,294,324^				> 1 Week^56,294^
***Social defeat stress*** Resident-intruder social defeat		EPM^71,89,90^		SP, FST, SPAT, olfactory habituation-dishabituation^89,310,325,326^	ASR^326^			>Month^310^
Witnessed social defeat	Contextual reminder^327^	EPM^328^		SP, FST, SAAT, tail suspension^327,328^				>1 Week^328^
Cage-within-cage resident-intruder social defeat								
***Predator stress*** Predator exposure stress	Contextual and cued reminders^95,329^	EPM, OF, LDB^95,97^	RAWM^330^		ASR^97^			>2 Weeks^95^
Predator-based psychosocial stress		EPM, OF^108^						
Predator scent stress		EPM^120,331^	MWM^120^		ASR^120,331^			>1 Week^120^

**Table 5:** Evaluation of reviewed animal models against validity criteria for animal models of human mental disorders^7^.

	Face validity: represents symptoms of the human disorder	Construct validity: represents the cellular and molecular mechanisms in the human patient (homologous constructs)	Predictive validity: demonstrates successful use of effective pharmacological treatments in human patients, discriminates between effective/ineffective treatments
***Physical stress*** Electric shock	^234,323^	^274^	Paroxetine, fluoxetine, sertraline^28,332,333^
Immobilization stress	^34,41^	^31,33,42^	Venlafaxine^334^
Underwater trauma	^44,45^	^48^	
Single prolonged stress	^53,324^	^61,62^	Paroxetine^335,336^
***Social defeat stress*** Resident-intruder social defeat	^89,307^	^72,91,312^	Fluoxetine, sertraline^264,337^
Witnessed social defeat	^313,328^	^74^	Fluoxetine^67^
Cage-within-cage resident-intruder social defeat	^68^	^338^	
***Predator stress*** Predator exposure stress	^96,97^	^98,339^	Fluoxetine^340^
Predator-based psychosocial stress	^101^	^110^	Sertraline^341^
Predator scent stress	^129,130^	^113,331^	Sertraline^342^

**Table 6:** Evaluation of reviewed animal models against Yehuda and Antelman’s criteria for animal models of PTSD^8^.

	Induces *biological* and *behavioral* sequelae of PTSD	Produces PTSD-like sequelae in a dose-dependent manner	Produces *biological* alterations that persist or become more pronounced over time	Induces bidirectional *biobehavioral* alterations	Produces interindividual variability in response
***Physical stress*** Electric shock	^234,323^	^28,224^	^323^	^332^	^28^
Immobilization stress	^34,41^	^30,343^	^344^		
Underwater trauma	^44,45^		^345^		^45^
Single prolonged stress	^53,324^		^61,62^		^54,286^
***Social defeat stress*** Resident-intruder social defeat	^89,307^	^346^	^72^	^326^	^73,90^
Witnessed social defeat	^313,328^		^20,67^		^74^
Cage-within-cage resident-intruder social defeat	^68^		^242^		
***Predator stress*** Predator exposure stress	^96,97^		^347^		^96,99^
Predator-based psychosocial stress	^101^		^110^		
Predator scent stress	^129,130^	^118^	^348^		^112,124^

Many behavioral tests were developed and validated in rats, and only later were adapted for mice. Rats were traditionally the species of choice in preclinical research for their performance in operant tasks and their larger size that facilitates application of invasive techniques, as well as toxicity tests of compounds. Mice, on the other hand, are advantageous for their ease of genetic modification, breeding, and group housing. Following the increasing use of mice, behavioral tests were translated into mouse versions, but with mixed success^131,132^. Therefore, the utility of mouse models is contingent on the availability of more behavioral tests that are optimized for use in mice.

More specific behavioral tests should be incorporated into batteries to address gaps in PTSD research. Since sucrose preference does not distinguish between affective response and motivation, more specific tests are needed such as facial reactivity analysis and measurements of reward-related ultrasonic vocalizations^133,134^. Other possible attention assessments that have not been conducted in the context of PTSD include the signal detection test with blank trials for sustained attention and multiple-choice serial reaction time test for sustained and selective attention^135^. The resident-intruder test has been used to assess irritability and aggression. However, this test is not listed in Table 2 because it incorporates the unclear stress effects of novel social interaction and could influence later behavioral time points.

One unrealized strength of rodent models of PTSD is in the discovery, development, and testing of effective pharmacological treatments. Historically, pharmaceutical companies did not invest in innovative drug discovery programs because modifying approved medications, particularly selective serotonin reuptake inhibitor (SSRI) antidepressants, was less time-consuming and more profitable^136^. While the SSRIs sertraline (Zoloft) and paroxetine (Paxil, Paxil CR, Brisdelle, and Pexeva), are the only medications approved for PTSD by the Food and Drug Administration (FDA) to date, off-label pharmacological treatments include fluoxetine (Prozac, Prozac Weekly, and Sarafem) and venlafaxine (Effexor XR)^137–140^. These drugs were tested in PTSD patients because of their effectiveness in treating depression. Many provide relief in patients, yet no medications have been approved to promote resilience. Therefore, pharmacotherapy is currently recommended as an adjunctive or next-line treatment to trauma-focused psychotherapy^139,141^. Interestingly, the two medications that are indicated for PTSD treatment, sertraline and paroxetine, and the two most promising drug candidates, ketamine and 3,4-methylenedioxymethamphetamine-assisted psychotherapy, did not emerge from basic research^142^. In addition, only some drugs have been tested in rodent models to study their targets and mechanisms of action (Table 5). One explanation for their mechanism is that medications may enhance psychotherapy’s efficacy to engage biological targets associated with recovery or resilience^142^. Alternatively, medications may distinguish the PTSD biological subtypes that respond to their targets^143,144^. Now that rodent models of PTSD are more validated and have been refined over the last 20 years, their use could elucidate human psychopathology and further reveal mechanisms driving recovery.

In rodents, as in humans, different traumas can cause different PTSD-like symptoms. Each with their advantages and disadvantages, there is no single model that serves all purposes. All reviewed animal models have phenomenological similarities to PTSD and satisfy face validity. Peripheral biologic correlates of PTSD have been evaluated for all stressors, generally meeting construct validity. For example, there are strong hypotheses regarding the neural mechanisms involved in PTSD, such as hyperactivity of the corticotropin-releasing hormone system, that have been verified in rodents^145,146^. However, because the biological basis of PTSD is not well understood, work needs to be done to define clear conditions for construct validity. Although preclinical research on traumatic stress has not yet resulted in drastic improvements in PTSD treatments, most models test pharmacological treatment options, providing them with predictive validity and potential for use in the development of clinical therapy. Given these results, more well-studied models such as electric shock and single prolonged stress may fit more criteria, but lack demonstration of multiplex behavioral outcomes in individual animals. While DSM-5, validity, and Yehuda and Antelman’s criteria each capture some of PTSD’s complexity, consideration of all three may be used to improve categorization of a rodent model’s trauma effects and inform stress paradigm selection. More accurately capturing the disorder in any individual model is critical, but overly limiting the types of models studied may be counterproductive in developing a comprehensive understanding of such a variable disorder.

## Challenges

### Symptom comorbidity

Symptom comorbidity complicates PTSD diagnosis and treatment. Although rodent models are useful for symptom analysis, there are limitations to generalizing stress in rodents to PTSD in humans since clinical diagnosis heavily relies on a patient interview rather than quantitative diagnostic measures. Individuals with PTSD are 80% more likely than those without PTSD to have symptom comorbidity. Symptoms such as avoidance, anhedonia, and exaggerated startle response may overlap with symptoms of other mental illnesses (e.g., depressive, bipolar, anxiety, or substance use disorders), making it difficult to attribute them to a specific PTSD model^6^. This creates challenges in distinguishing animal models of PTSD from other psychiatric illness models. The criteria used in the development and study of PTSD models will continue to evolve as scientific understanding of PTSD grows and the DSM is updated. Nevertheless, longitudinal studies are used to develop animal models with relevant PTSD symptom domains and to test causal factors in symptom development.

### Anthropomorphism

The anthropomorphizing that can occur when uniquely human characteristics are attributed to animals is a danger of animal models and complicates their translatability. Many diagnostic criteria of mental disorders are subjective, involving thoughts, memories, and their interpretations. Such criteria as intrusive thoughts, emotional blunting, and cognitive distortion cannot be measured in animals without the danger of excessive anthropomorphism. Therefore, research is limited to observable behaviors with quantifiable measures, many of which are detailed in Table 2. However, even these measures have limitations, since rodent behavioral tests often do not directly correlate to human tests. The urgency to perform “translational” work can cause research groups to exaggerate a treatment’s effects and scientific foundation, ultimately hindering treatment development. Prolonged exposure, for example, is a cognitive behavioral therapy that only works for some patients and may not be based on fear extinction, yet extinction learning is so robust and explainable in animals that it is used to justify the treatment’s mechanism^147^. From the “translational” research opportunity and ease of modeling fear acquisition and extinction, this single aspect of PTSD has become more significant than it is clinically^148^. Anthropomorphizing can also result in data overinterpretation. For example, rodents that demonstrate escape or freezing behavior have been referred to as anxious rather than, more appropriately, exhibiting anxiety-related or anxiety-like behavior^149^. Further, this anthropomorphizing can lead to misleading interpretations of behavior, as it has been shown that behaviors like freezing are not simple quantitative measures of fear or anxiety as they are often utilized^150^. Therefore, to optimize the study of PTSD, rodent models should be empirically based, without anthropomorphic inference.

### Non-standardized experimental designs

Experimental designs are often not standardized across laboratories, complicating inter-laboratory comparison and result replication. Studies differ in their methods of trauma exposure, behavioral test batteries, and data analysis. This variability is exacerbated through differences in experimenter sex, rodent sex, species/strain/stock, age, housing and noise conditions (e.g., stressed and control animals caged separately/together, individually ventilated cages), food, incubation time between trauma and testing, acclimation time to the testing room and its light intensity, location of trauma and testing rooms, transportation to the testing room, subject location during trauma and testing of other subjects, specific tests conducted and their order, test duration, and test timing (light or dark cycle). All of these factors have a non-trivial effect on results and illustrate the often-underestimated importance of attention to detail.

### Insufficient reporting

Many publications have insufficiently reported experimental details, rendering them not fit-for-purpose and limiting their value to researchers, doctors, and policymakers^151^. This challenge is compounded by concerns of positive publication bias, which favors the reporting of positive results (data supporting an alternative hypothesis) and can lead to spurious claims, wasteful experimentation, and reduced meta-analysis validity. Some journals (e.g., Journal of Negative Results in BioMedicine, Journal of Pharmaceutical Negative Results, Nature Negative Results section) compensate publication bias by exclusively publishing negative results. Favoring negative results, however, can also introduce bias^152^. Therefore, publishing criteria should focus on a study’s design and statistical power, regardless of its outcome. Guidelines for planning and reporting animal experiments (e.g., Gold Standard Publication Checklist, Animal Research: Reporting of In Vivo Experiments, and Planning Research and Experimental Procedures on Animals: Recommendations for Excellence) have been established, but have not yet been widely adopted^153–155^. The lack of reproducibility in preclinical research may contribute to the failure of drugs in clinical trials^156^.

### Ethical complications

Effective rodent models of PTSD utilize stressors that exceed the animal’s homeostatic regulatory capacity, but ethical complications remain. Studies are expected to minimize rodent suffering, improve human health, and advance general scientific knowledge. Since exposure to a significant trauma is required, emphasizing reduction in animal distress is counterproductive in PTSD models. This may result in minimizing exposure type or severity such that the model’s efficacy and scientific value is compromised^10,157^. Predator scent stress, for example, developed in response to safeguards limiting predator-prey interactions, is less severe and may not as effectively induce the PTSD-like phenotype as predator exposure. Therefore, stressful experiences that are within a rodent’s coping capacity should be distinguished from traumatic experiences that are beyond its coping capacity. Investigators are encouraged to collaborate with ethical committees to ensure that their selected exposure is relevant to PTSD and maximizes the probability of clinically significant findings.

## Future directions

While it is apparent that well-established PTSD animal models are available, refining behavioral and neurobiological understanding of these models is still needed. Future directions include study of (1) individual variability and behavioral test batteries; (2) sex differences; (3) strain and stock differences; (4) early life stress effects; (5) biomarkers; as well as use of (6) stringent success criteria for drug development; (7) Research Domain Criteria (RDoC); (8) technological advances; and (9) cross-species comparisons.

### Individual variability and behavioral test batteries

Many studies regard groups of animals under a condition as homogeneous, overlooking individual variability, and rely on a small number of tests with singular time points. Some models (e.g., immobilization stress, predator-based psychosocial stress) create a ubiquitous response in which all exposed animals present PTSD-like symptoms without the response variability inherent to human PTSD. Trends in variability have been described as “bidirectional” (Table 6), but with different meanings across research groups. Bidirectional expression has been used to mean both the concurrence or alternation between increased (i.e., intrusive re-experiencing, hyperarousal) and decreased (i.e., avoidance, numbing) responsiveness across different behavioral tests or subgroupings of animals within an experimental group. For example, a model could satisfy Yehuda and Antelman’s bidirectional criterion by showing that a group of animals display hyperarousal in ASR and anhedonia in sucrose preference or that stressed subgroups show opposite responses to the same stimulus. When only a subgroup is expected to display long-term PTSD-like phenotypes, analyzing group level statistics based on exposed and control groups can lead to weaker statistical power and would make results harder to translate. While animal behavioral studies often employ few tests post-exposure to detect symptoms, test batteries covering multiple diagnostic criteria can achieve a more reliable profiling of individual animals. With multiple tests and time points, behavioral changes measured before and after stress could provide insight into the direct effects of stress over time, controlling for subject variability. Interpreting results in a manner similar to human diagnosis (e.g., cut-off behavioral criteria^96^, median split criteria^119^, behavioral profiling^48^, or clustering methods) then allows for the classification of susceptible and resilient trauma-exposed individuals. By researchers considering individual variability, treatments can be evaluated on whether they decrease the proportion of maladapted animals instead of their effect on symptom severity.

### Sex differences

Sex differences are another area of variability that has been poorly studied (Table 4). PTSD was introduced into the DSM-III following the high prevalence of male Vietnam veterans seeking treatment for posttrauma symptoms^158^. All psychiatric disorders display sex differences, yet male rodents have been predominantly studied given the historical perspective of past publications^159^. Over-reliance on male animals and cells conceals sex differences and may contribute to the increase of non-translationality in preclinical research^156^. Due to the push to address sex differences, in consideration of females’ higher prevalence of PTSD and adverse drug reactions, preclinical studies are now studying sex differences more readily^6,160^. Noting discrepancies in diagnostic criteria between the DSM-IV, DSM-5, and 10th Revision of the International Classification of Diseases (ICD-10), studies suggest that sex differences in symptom endorsement may make females more likely to meet DSM-5 and ICD-10 criteria for PTSD. Females were more likely to endorse intrusive thoughts, avoidance of external reminders, emotional blunting, exaggerated startle response, and sleep disturbance, while males were more likely to endorse aggressive behavior symptoms^161,162^. Sex differences in coping strategies, possibly mediated by differential endocrine responses (e.g., cortisol and oxytocin), have also been proposed^163^. Furthermore, human and rodent findings have suggested that differences in interactions between gonadal hormones testosterone or estrogen with the HPA axis or hippocampus contribute to the increased disorder risk among females^164–166^. Evaluations of behavioral effects of estrous cycle phase in female rodents observed trait and strain differences in test performance stability across phases, concluding that choices of behavioral paradigms, testing conditions, and genetic backgrounds are critical to controlling for hormonal effects^167^. However, hormone variation should be monitored as it could confound data interpretation or behavioral phenotyping performed blind to estrous cycle phase. In addition, studies should be designed with an adequate sample size to homogenize the estrous phase distribution. Further research is needed to understand the effects of hormones on PTSD and to develop hormone-sensitive treatments.

### Strain and stock differences

Similar to humans, rodent genetic background modulates stress susceptibility and phenotype variation, yet strain and stock differences have not been sufficiently characterized. Inbred strains of mice (e.g., C57BL/6, BALB/c) and rats (e.g., Lewis, Fischer), genetically homogenous, are used to minimize intrastrain differences. In contrast, outbred stocks of mice (e.g., Swiss, CD-1) and rats (e.g., Long-Evans, Sprague-Dawley), genetically heterogenous, are used to model the variability of human populations. The C57Bl/6 strain is reported to be more resilient to early life stress compared to strains such as the anxious BALB/c strain^168,169^. In addition, strain and stock differences in baseline behavior and psychotropic treatment response have been identified^170,171^. Therefore, rodent strain/stock, vendor, and colony should be considered across experiments within a study to better control experimental conditions and increase result reproducibility. Further research may reveal phenotypic behavioral differences with underlying genetic bases relevant to PTSD susceptibility.

### Early life stress effects

Although early life stress, such as childhood abuse, increases vulnerability to psychiatric disorders later in life, this relationship is not clearly understood^172^. To better understand this relationship, early life stress is modeled in rodents through interventions in mother-pup interaction time periods (e.g., early handling for 3–15 min, repeated maternal separation for 1–8 h, single maternal separation for 24 h), the quantity and/or quality of maternal care (e.g., repeated cross-fostering, naturally occurring differences in maternal care, impoverished postnatal environment), and pharmacology (e.g., postnatal glucocorticoids, postnatal lipopolysaccharide)^173,174^. Since the brain’s neurocircuitry matures from adolescence to early adulthood, trauma response may change over time^175^. In addition to effects of early life stress on brain and behavior over the developmental course, open research areas include identification of gene-environment interactions, epigenetic processes, sensitive periods, methods for disorder prevention or reversal, and beneficial effects. Different hypotheses exist to explain the interaction between early and adult stress in mental disorder susceptibility. Whereas multiple hit models, such as the cumulative-(diathesis-)stress hypothesis, state that disease risk increases with more adversity, the match/mismatch hypothesis states that risk deceases when early and adult environments match (stress inoculation), but increases upon mismatch^176,177^. Similarly, the differential susceptibility to environmental influence (“for-better-and-for-worse”) hypothesis states that individuals carrying alleles that are reactive to the environment display heightened sensitivity to disease risk in stressful contexts, but also to beneficial effects in supportive conditions^178^. Integrating hypotheses and considering individual differences in early programming sensitivity, the three-hit concept of vulnerability and resilience incorporates genetic predisposition, early life environment, and later life environment^179,180^. Additional assessments across the life span should be conducted to evaluate these hypotheses and clarify the relationship between maltreatment in early life and mental illness in adulthood.

### Biomarkers

Potential PTSD biomarkers have been identified, but the topic remains underdeveloped as there are no biomarkers of susceptibility, disease, or therapy in clinical use. An individual’s susceptibility to developing PTSD can be evaluated before trauma through risk markers for primary prevention, to prevent exposure, and early after trauma through risk markers for secondary prevention, to begin preventative therapy before symptom expression. Reported primary risk markers in humans include: (1) high fear response to a single 35% CO_2_ inhalation challenge^181^; (2) gene × environment interactions (e.g., FK506 binding protein 5 (FKBP5) gene × childhood trauma)^182^; (3) HPA-axis regulators^183^; (4) frequent nightmares (possibly related to the hampered fear extinction memory consolidation associated with REM sleep)^184^; and (5) low hippocampal volume^185^. Few secondary risk markers, apart from increased heart rate^186^, have been reported because of sample size and prospective design limitations. Prominent disease diagnostic markers include HPA-axis dysregulation^187^, FKBP5 expression^188^, sympathetic nervous system hyperreactivity^189^, low hippocampal volume^190^, and high amygdala activity^191^. Use of biomarkers as inclusion or exclusion criteria for patient enrollment into clinical studies increases a drug candidate’s likelihood of FDA approval^192^. However, few PTSD therapy stratification/selection markers, used to predict treatment responses and stratify patients by therapy responder types, or progress markers, used to monitor therapy responses, have been determined. The field’s attempts to identify PTSD biomarkers has not resulted in treatments, so the aim that animal models will provide targets that will inform treatments has not yet been accomplished^142^. This is because biomarkers are currently limited by their interdependence, contribution to other disorders, inconsistent effect distribution, and small effect^146^. It is, therefore, important to determine how to use animal models to successfully translate findings. With multimodal study designs, identification of biomarkers that are homologous across species and play similar roles in the model and clinical condition will compliment behavioral measures to inform clinical trials of diagnostic and treatment targets. For reviews of PTSD biomarkers, see DePierro and Schmidt^142,193^.

### Stringent success criteria

Implementing stringent success criteria into the preclinical stage of testing may improve upon the traditional drug development approach of expediting treatments into clinical trials to collect human data as early as possible. The unmet need for PTSD treatments may have reduced regulatory restrictions to their development and allowed drugs with suboptimal preclinical validation to enter clinical trials. Consequently, central nervous system drug candidates now have the second lowest success rate among therapeutic areas and take longer to bring to market^192,194^. The majority of drugs that showed initial promise in animal models failed in phases II and III of clinical development due to a lack of efficacy^195–198^. For example, Bionomics’ BNC210, a negative allosteric modulator of the α7 nicotinic acetylcholine receptor, failed to significantly improve symptoms compared to placebo in a Phase II PTSD trial because of insufficient exposure (bioavailability)^199^. Target site exposure, target binding, and expression of functional pharmacological activity (the “three pillars of survival”) all determine the likelihood of candidate survival in Phase II and progression to Phase III trials^200^. This underscores the importance of acquiring preclinical pharmacokinetic and pharmacodynamic data to test drug mechanisms and success. In response to the high unsustainable cost of clinical trials, pharmaceutical companies, including Bionomics, have downscaled, outsourced, or closed their neuroscience research programs^201–203^. The FDA estimates that a 10% improvement in failure prediction before clinical trials could save $100 million in development costs per drug^204^. Stringent success criteria would improve failure prediction by requiring more preclinical validation, thus shifting risk from the clinical to the preclinical stage. This may then decrease drug development costs, times to market, and prices to the public^151^.

### Research domain criteria

PTSD is a heterogeneous disorder and the field is shifting focus from generalized diagnostic criteria toward targeting specific symptom domains. In parallel to disease-specific DSM-5 criteria, the US National Institute of Mental Health’s RDoC is a framework for subject characterization based on degrees of dysfunctions in psychological/biological systems. Therefore, the RDoC assumes that there is a dimensional continuum between health and pathology. The RDoC matrix consists of dimensional psychological constructs (or concepts), grouped into higher-level domains of human behavior and functioning, and distinguished by the units of analysis used to investigate constructs. Continuously evolving from more construct validity and utility data, the RDoC matrix currently has five domains (negative valence, positive valence, cognitive, social processes, arousal and regulatory systems) and eight units of analysis (genes, molecules, cells, circuits, physiology, behavior, self-report, paradigms)^205^. Additional domains that are relevant to PTSD and psychiatric research have been proposed, namely stress and emotional regulation and maintenance of consciousness^206^. Intended to inform future diagnostic systems, RDoC integrates multiple measures for a comprehensive understanding of matrix constructs and, consequently, their corresponding symptoms. Investigators should align studies to RDoC as it encourages scientists to seek beyond observable results to neurobiological measures. Using a more stringent criteria for what we consider as a rodent model based on the criteria discussed can improve the translatability of results.

### Technological advances

Utilizing technological advances in neuroimaging and genetic manipulation in PTSD research is promising as it allows for longitudinal assessments and investigations of circuits governing PTSD etiology. In clinical practice, neuroimaging, specifically structural, is mostly used to exclude brain pathology from the cause of psychiatric symptoms. Since diagnosis is subjective, quantitative clinical measures are needed to validate the current qualitative criteria and give preclinical researchers confidence that they are modeling PTSD, rather than genetic variability. Recent technological advances may be employed to achieve this goal and improve information flow between the two fields. fMRI, for example, measures blood-oxygenation-level dependent signal as an indirect indicator of neuronal activity. It is a powerful noninvasive measure of systems-level brain function and has a higher spatial resolution than other noninvasive modalities such as electroencephalography. MRI has been used to demonstrate altered fractional anisotropy, focal neural activity, functional connectivity, as well as focal atrophy of gray matter^207^. Awake rat rsfMRI avoids confounding factors from anesthetics and can be correlated with behavioral measures to study functional networks implicated in stress response, discover new regions of interest, and define endophenotypes of susceptibility^208^. Since fMRI can be applied to both animals and humans, findings may be translated across species and the conservation of neural circuits may be compared^209^. Stress signaling pathways can also be selectively stimulated via optogenetics and chemogenetics (or pharmacogenetics). Optogenetics enables manipulation of neuronal activity in stress circuits through activation of light-sensitive proteins, such as microbial opsins, that are selectively inserted into intact living mammalian neurons. Using this technology, researchers can control cell firing patterns with high tissue specificity and temporal precision to study the causal relationship between circuits and behavior in PTSD^210^. In chemogenetics, instead of with light, cell populations and neural circuits are modulated by the injection of biologically inert ligands that activate engineered receptors. Designer Receptors Exclusively Activated by Designer Drugs are widely used in chemogenetics and act through G-protein coupled signaling cascades. Chemogenetics has a lower temporal resolution, but is relatively noninvasive compared to optogenetics, which requires fiber-optic implants^211^. Integrated with fMRI, these technologies can map the downstream effects of local manipulations on global brain activity^212–214^. The neurobiology of stress can also be studied through diffusion tensor imaging^215^, magnetoencephalography^216^, calcium-based fiber photometry^217,218^, and molecular fMRI^214^. Combined with fMRI, these multimodal methods allow researchers to collect complementary and multi-dimensional information across spatiotemporal scales on the same animal. Implementation of these tools in studies of PTSD animal models and integration with established techniques in behavioral analysis, electrophysiology, and pharmacology can unveil progressive changes after stress exposure and susceptibility endophenotypes. While reliance on qualitative assessment is still standard, the shift towards multimodal methods will allow for the determination of quantitative PTSD biomarkers that are predictive of qualitative criteria used to assess PTSD prevalence. This goal will likely require increased collaboration between clinical and preclinical researchers to be achieved.

### Cross-species comparisons

Cross-species comparisons of stress responses over the phylogenetic scale, ranging from rodents to animals in the genus Mustela (e.g., weasels, ferrets, minks) and nonhuman primates, are required to truly understand the preservation of PTSD features. Neurobiological results from rodent studies have already shown relevance in other species, but with mild stress models^219,220^. For example, both rodent and primate research has identified brain regions, such as the bed nucleus of the stria terminalis (BNST), amygdala, prefrontal cortex, cingulate cortex, and hippocampus, that regulate stress-related behavioral mechanisms and are dysfunctional in PTSD patients^146^. The BNST, in particular, has been investigated for its role in fear memory neural circuitry^221^. In mice, optogenetic photoinhibition of neurons projecting between the BNST and ventral tegmental area during electric shock stress decreased freezing in the exposure context and closed arm entries in the EPM^222^. Consequently, the BNST was investigated in rhesus monkeys and its intrinsic functional connectivity with the central nucleus of the amygdala was found to be correlated with behavioral responses to no-eye-contact human intruder stress^223^. A possible translational roadmap for studying PTSD is to combine mechanistic and behavioral measures from rodents with multimodal neuroimaging measures from nonhuman primates and humans. Cross-species comparisons such as these show promise to elucidating the clinical relevance of stress-induced cellular, molecular, and microstructural discoveries in rodents.

## Conclusion

**Fig. 1:**
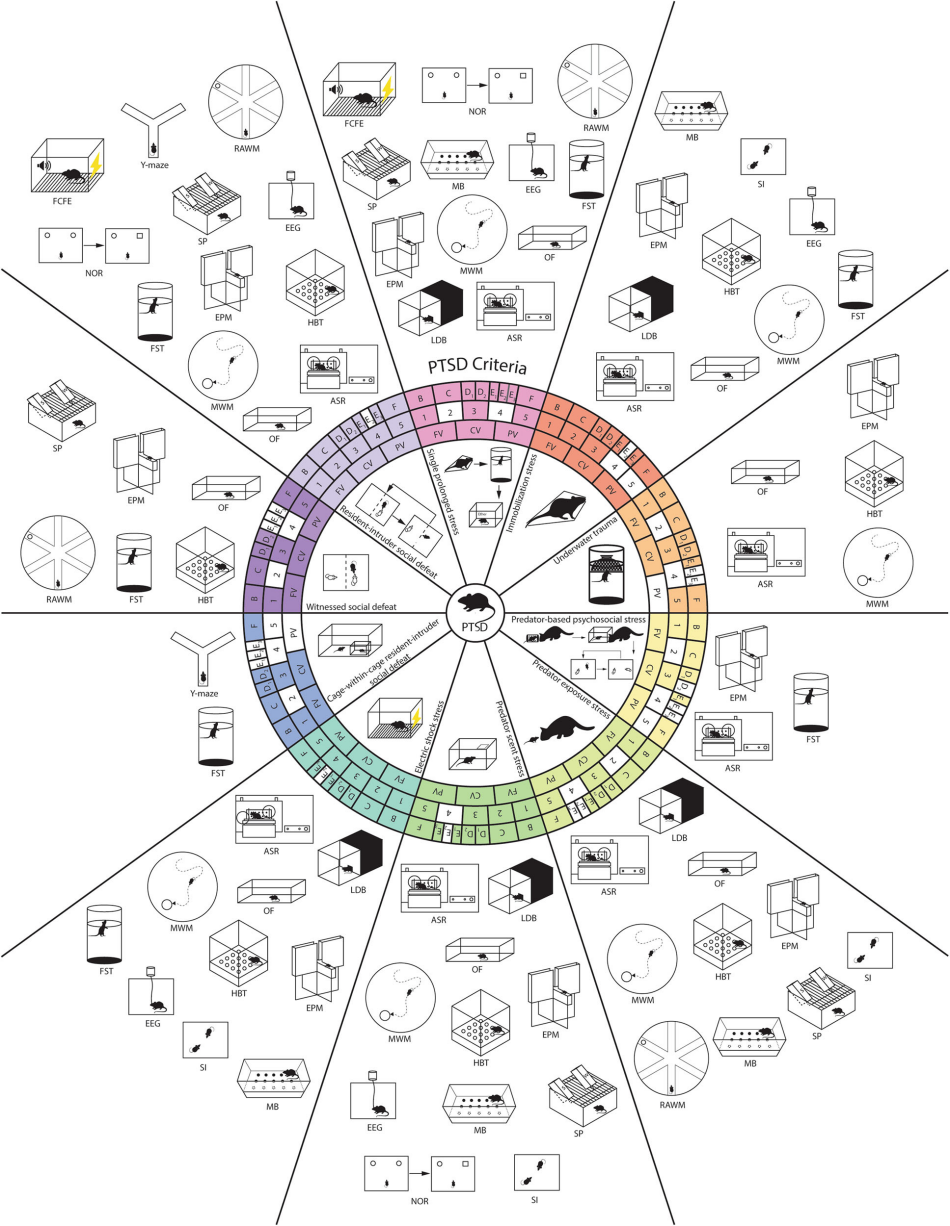
Superimposition of reviewed animal models against validity criteria for animal models of human mental disorders (inner circle), Yehuda and Antelman’s criteria for animal models of PTSD (middle circle), and DSM-5 criteria for PTSD (outer circle). Validity criteria abbreviations: face validity (FV), construct validity (CV), predictive validity (PV). Yehuda and Antelman’s criteria abbreviations: biological and behavioral sequelae of PTSD (1), dose-dependent (2), lasting symptoms (3), bidirectional (4), interindividual variability (5). DSM-5 criteria abbreviations: physiological reactions to trauma reminders (B), increased avoidance (C), cognitive alterations (D_1_), mood alterations (D_2_), increased arousal (E_1_), concentration problems (E_2_), sleep disturbance (E_3_), lasting symptoms (F). Behavioral test abbreviations: elevated plus maze (EPM), open field (OF), light-dark box (LDB), Morris water maze (MWM), radial arm water maze (RAWM), fear conditioning / fear extinction (FCFE), novel object recognition (NOR), social interaction (SI), forced swim test (FST), sucrose preference (SP), marble burying (MB), acoustic startle response (ASR), electroencephalogram (EEG). White: not reported. Shaded: reported.

Since a variety of traumas, producing specific symptoms, can trigger PTSD, a single rodent model will not perfectly capture a human individual’s disorder complexity. While individual animal models can replicate lack of coping and ineffective adaptation to stress, this does not necessarily represent coverage of human PTSD variability. This shortcoming is in part rooted in the modeling of general trauma conditions such as life threats, while modeling effects of particular stimuli (e.g., burning odors, sounds of gunfire) or cognitive assessments (e.g., helplessness, self-blame, coping strategy) of the general trauma condition is not further explored. As studies of animal models continue to improve, incorporation of experimental designs that can highlight the effect of specific features of a trauma or drive specific coping strategies will improve animal models’ representation of human PTSD variability. Similarly, more objective measures are needed to evaluate a rodent’s inability to self-regulate, also a cognitive aspect of responses to anxiogenic stimuli, to assess aspects of human PTSD variability further in preclinical studies. This, along with the availability of physical, social, and psychological rodent stressors, varying in length and intensity, will improve translation of results to comparable clinical cases. Each model’s strengths and limitations, reviewed here, should be considered for fit-for-purpose stress paradigm selection, hypothesis testing, and experimental design. For example, consideration of the DSM-5 risk and prognostic factors for PTSD in animal models (e.g., trauma reminders, female subjects, and early life stress) are likely to better replicate the human condition in patients with similar features. When evaluating paradigms, judging rodent models based on one set of criteria could be misleading because each focuses on different constructs. DSM-5 criteria, designed to facilitate diagnosis of human mental disorders, are used to compare stressors by their representation of PTSD symptomatology. Expanding the comparison to etiology and treatment response, validity criteria are used for animal models of human mental disorders. Yehuda and Antelman’s criteria, more specific, are used for animal models of PTSD and include factors that are essential to the induction of PTSD-like phenotypes. Therefore, all three criteria should be superimposed for the optimal reflection of a stressor’s relevance to PTSD (Fig. 1). As only a minority of individuals who experience traumatic event(s) develop the disorder, it is important to investigate animals’ individual variability in stress response and subsequent development of PTSD-like behaviors to distinguish susceptibility. It is also important to remember that the goal of this research is to expand knowledge and improve the clinical treatment of PTSD. Despite the large financial investment and the thousands of papers published annually, no treatments have been approved for PTSD since Paxil in 2000. With the incorporation of the directions discussed, rodent models of PTSD can provide more utility and translational impact as they lead to shifts in practice.
